# Induction of Multiple Immune Regulatory Pathways with Differential Impact in HCV/HIV Coinfection

**DOI:** 10.3389/fimmu.2014.00265

**Published:** 2014-07-08

**Authors:** Hyosun Cho, Masahiro Kikuchi, Yun Li, Nobuhiro Nakamoto, Valerianna K. Amorosa, Mary E. Valiga, Kyong-Mi Chang

**Affiliations:** ^1^Philadelphia VAMC, Philadelphia, PA, USA; ^2^Department of Medicine, University of Pennsylvania School of Medicine, Philadelphia, PA, USA; ^3^Duksung Women’s University, Seoul, South Korea

**Keywords:** Tregs, PD-1, CTLA-4, immune pathogenesis, HCV, HIV, coinfection, FoxP3

## Abstract

Persistent viral infections including HCV, HBV, and HIV are associated with increased immune regulatory pathways including the extrinsic FoxP3+CD4+ regulatory T cells (Tregs) and intrinsic inhibitory pathways such as programed death-1 (PD-1) and cytotoxic T lymphocyte antigen-4 (CTLA-4) with potentially reversible suppression of antiviral effector T cells (1–12). Immunological consequences of viral coinfections relative to these immune regulatory pathways and their interplay are not well-defined. In this study, we examined the frequency, phenotype, and effector function of circulating T cell subsets in patients with chronic HCV and/or HIV infection, hypothesizing that HCV/HIV coinfection will result in greater immune dysregulation with pathogenetic consequences (13, 14). We show that multiple T cell inhibitory pathways are induced in HCV/HIV coinfection including FoxP3+ Tregs, PD-1, and CTLA-4 in inverse association with overall CD4 T cell frequency but not with liver function or HCV RNA titers. The inverse association between CD4 T cell frequency and their FoxP3, PD-1, or CTLA-4 expression remained significant in all subjects combined regardless of HCV and/or HIV infection, suggesting a global homeostatic mechanism to maintain immune regulation relative to CD4 T cell frequency. PD-1 blockade rescued T cell responses to HIV but not HCV without significant impact by CTLA-4 blockade *in vitro*. Collectively, these findings highlight complex immune interactions in viral coinfections and differential regulatory pathways influencing virus-specific T cells that are relevant in immunotherapeutic development.

## Introduction

FoxP3+CD4+ regulatory T cells (FoxP3+ Tregs) play a key role in mediating immune tolerance to self and non-self antigens including viral pathogens (15). Programed death-1 (PD-1) and cytotoxic T lymphocyte antigen-4 (CTLA-4) are receptors in CD28 family of co-stimulatory molecules that are induced by T cell activation and provide inhibitory signals to T cells (16). There are further cross-talks between the “extrinsic” regulatory T cells (Tregs) and “intrinsic” T cell co-stimulatory pathways (17, 18). In chronic viral infection including HCV, increased circulating frequency of CD4+FoxP3+ Tregs as well as increased PD-1 and/or CTLA-4 expression on virus-specific T cells have been reported (6, 7). Similarly, HIV persists with dysfunctional antiviral effector T cells with increased PD-1 and/or CTLA-4 expression (3, 19, 20). HIV infection has also been associated with increased FoxP3+ Tregs in peripheral CD4 T cell compartment despite HIV-associated CD4 T cell loss in some (21–25) although not all studies (26–29). Furthermore, PD-1 and CTLA-4 expression in antiviral T cells correlated with HIV viral load and CD4 T cell count in HIV-monoinfected patients (3–5).

Further immune dysregulation may occur in HCV/HIV coinfection due to HCV-mediated immune activation and HIV-associated CD4 T cell loss (24, 30). Such interactions may have clinical implications, given worsened outcomes in HCV/HIV-coinfected patients (13, 14). However, potential interplays between FoxP3+ Tregs and intrinsic T cell co-stimulatory pathways in viral infections have not been well-defined. Moreover, it remains unclear how they impact antiviral effector T cell response as well as HCV and/or HIV-associated outcomes. These are relevant considerations in immunotherapeutic development for chronic viral infection (2–12, 31) as well as cancer patients with concurrent viral infections (32).

In this study, we show that FoxP3+ Tregs are preserved in the CD4 T cell compartment of HCV/HIV-coinfected patients in direct association with PD-1 and CTLA-4 expression on CD4 T cells but not with liver function measures or HIV viremia. Furthermore, FoxP3, PD-1, CTLA-4, CD28, and CD127 expression showed significant associations with the overall CD4 T cell frequency, suggesting a global homeostatic mechanism to maintain immune regulation as the size of CD4 T cell compartment changes. Finally, HIV-specific effector T cells were not only more detectable in peripheral blood and but also more responsive to PD-1 blockade *in vitro*, compared to HCV-specific T cells.

## Materials and Methods

### Study subjects

Study subjects were recruited from the Hepatology and Infectious Diseases clinics in the Philadelphia Veterans Affairs Medical Center and the Hospital of the University of Pennsylvania with informed consent approved by the institutional review board: HCV-monoinfection (HCV, *n* = 20), chronic HCV/HIV coinfection (HCV/HIV, *n* = 23), chronic HIV-monoinfection (HIV, *n* = 21), and seronegative controls (NC, *n* = 17). Exclusion criteria included: active immunosuppressive therapy, active antiviral therapy for HCV infection, HBV infection, autoimmune liver disease, or significant medical comorbidities that preclude blood draws or informed consent. Chronic HCV infection was defined by persistent HCV viremia by quantitative RT-PCR by Roche COBAS or TaqMan assays (Roche Diagnostics, Indianapolis, IN, USA) in HCV-seropositive persons without acute hepatitis. HIV infection was defined by detection of HIV Ab and/or HIV viral load. As shown in Table 1, most patients were males in their 50s consistent with our local patient population. Clinical liver-associated parameters (e.g., bilirubin, albumin, platelet count, prothrombin time INR, AST Platelet Ratio Index APRI) were largely preserved, although sALT activity was greater in HCV and HCV/HIV groups compared to HIV-monoinfected group. Majority of HIV-infected patients were on anti-retroviral therapy and displayed HIV viral load below 50 IU/ml.

**Table 1:** **Patient groups**.

	Chronic HCV	Chronic HCV/HIV	Chronic HIV	*P*-values***
	C	I/C	I	
	*n* = 20	*n* = 23	*n* = 21	
Sex (M:F)	19:1	23:1	22:0	NA
Age (years)*	55	55	48	0.13
SALT (IU/ml)*	47	50	27	<0.0001
Total bilirubin (mg/dl)*	0.7	1.0	0.7	0.16
Albumin (g/dl)*	4.4	4.2	4.5	0.077
Platelets (10^3^/mm^3^)*	197	205	201	0.85
INR*	1	1	1	0.67
APRI*	0.6	0.6	0.3	<0.0001
HCV genotype 1 (%)	85%	96%	NA	NA
median HCV RNA (IU/ml)	2,750,000	1,210,000	NA	0.98
HAART	NA	87%	95%	0.61
Time from HIV diagnosis*	NA	13 years	12 years	0.75
HIV viral load <50	NA	83%	61%	0.1
CD4 count**	NA	407	586	0.043

*Median values.

**CD4 count in 16 HCV/HIV and 15 HIV patients peformed within 1 month of immune analyses among HIV-infected patients.

***P-values by non-parametric Kruskal–Wallis for three groups and Mann–Whitney *U* for two groups.

*NA (not applicable)*.

### Isolation of peripheral blood mononuclear cells

Peripheral blood mononuclear cell (PBMC) were isolated by Ficoll-Histopaque (Sigma Chemical Co., St Louis, MO, USA) density centrifugation and used directly or cryopreserved as previously described (33).

### Peptides and HLA class I tetramers

HCV-specific T cell response was measured using a pool of 105 overlapping 15mer peptides spanning the entire HCV NS3 derived from HCV-H genotype 1a (34). HIV-specific T cell response was measured using a pool of 124 overlapping 15mer peptides spanning the entire HIV Gag (generously provided by the AIDS Research and Reference Reagent Program, Division of AIDS, NIAID, NIH) (35). T cell response to influenza virus was examined using 56 overlapping 15mer peptides spanning the conserved matrix M1 protein (residues 1–252) based on the human A/PR/8/34 (H1N1) virus. For HLA-A2+ subjects, the following peptides were synthesized for antigenic stimulation and tetramer synthesis as described previously (6, 7): (i) HCV NS3 1073 (CINGVCWTV), NS3 1406 (KLVALGINAV), and NS5B 2594 (ALYDVVSKL); (ii) HIV Gag (SLYNTVATL); (iii) influenza matrix (GILGFVFTL); (iv) EBV BMLF1 (GLCTLVAML); (v) CMV pp65 (NLVPMVATV).

### Fluorescent antibodies

All monoclonal antibodies were purchased from BD Bioscience (San Jose, CA, USA) except for anti-Foxp3 from eBioscience (San Diego, CA, USA). Foxp3 and CTLA-4 expression was examined using FITC-labeled αFoxp3 (clone PCH101, eBioscience) and PE-labeled αCTLA-4 (αCD152; clone BNI3, BD). Dead cells were excluded with 7AAD.

### Immunophenotyping by flow cytometry

Cells were stained by fluorescent antibodies as per manufacturer’s instructions with freshly isolated PBMC used directly *ex vivo* (36). Events were acquired with a FACSCanto (Becton Dickinson, San Jose, CA, USA) and analyzed with FlowJo (Tree Star Inc., Ashland, OR, USA), gating on live lymphoid cells based on scatter characteristics and viability staining (7AAD by Biolegend or Aqua LIVE/DEAD L34957 by Invitrogen) and single color compensation controls. Intracellular FoxP3 and CTLA-4 expression was detected after permeabilization with cutoffs determined by isotype controls (Foxp3: 99.9%, CTLA-4: 99.9%) as previously described (6, 36).

The frequency of FoxP3+CD4+ Tregs (%Foxp3+CD4+/lymphocytes) in total lymphocytes was measured as percentage of FoxP3+CD4+ T cells in live lymphocyte gate. FoxP3+ Treg frequency in CD4 T cells (%FoxP3+/CD4 T cells) was measured as percentage of FoxP3+ cells within CD4+ live lymphocytes. Similar strategies were used to define circulating frequency of CD4+ or CD8+ T cells expressing PD-1, CTLA-4, or CD28+ T cells in total, CD4+ or CD8+ live lymphocytes.

### Analysis of virus-specific T cell IFNγ and TNFα production in short term T cell line

PBL (2 × 10^6^ cells/ml/well in 24-well plate) were: (1) stimulated with media, overlapping 15mer peptide pools (HCV NS3, HIV Gag, or influenza matrix at 2 iM per peptide) on day 0; (2) supplemented by fresh media with rIL-2 (100 IU/ml) on day 3 or 4; (3) examined by antigen-specific TNFα and IFNγ production on day 7 by intracellular cytokine staining as previously described (7).

### Analysis of antigen-specific T cell expansion and effector function in the presence or absence of PD-1 and/or CTLA-4 blockade

PBL (2 × 10^6^ cells/ml/well in 24-well plate) were stimulated with overlapping HCV NS3, HIV Gag, or influenza matrix 15mer peptide pools (2 μM) in the presence of isotype control, aPD-L1 (clone 29E.2A3.C6), aCTLA-4 (clone BNI3; BD), or both aPD-L1 and aCTLA-4 (10 μg/ml for each mAb) as previously described (7). Cell cultures were stimulated with rIL-2 (100 IU/ml) on day 3 or 4 and examined for cytokine production by flow cytometry on day 7 after 6 h stimulation with and without HCV, HIV, or Flu peptides in the presence of brefeldin A (10 μg/ml) as previously described (7). Virus-specific responses were calculated by subtracting the cytokine responses in media control samples from peptide-stimulated samples during the intracellular cytokine staining. A positive response to blockade was defined as antigen-specific response that was greater than the isotype control condition by at least a standard deviation of the all background responses (0.36%).

### Cytokine analysis of FoxP3+ and FoxP3− T cells in PBMC *ex vivo*

PBL (2 × 10^6^ cells/ml/well in 24-well plate) were stimulated with or without overlapping HCV NS3, HIV Gag, influenza matrix 15mer peptide pools (2 μM) or 10 ng/ml phorbol 12-myristate 13-acetate (PMA; Sigma Chemical Co.), 200 ng/ml ionomycin (Sigma Chemical Co.) in the presence of brefeldin A (10 μg/ml) and FoxP3, and intracellular cytokine staining was performed after 5.5 h with a Cytofix/Cytoperm kit (BD Pharmingen) as previously described (36).

### IFNγ ELISPOT assay

IFNγ Elispot assays were performed with 200,000 PBMCs per well stimulated in triplicates by overlapping pHCV NS3, pHIV Gag, or pFlu matrix 15mer peptide pools (2 mM) or media alone. After 45 h, plates were developed and IFNg spot forming units (SFUs) counted by an ELISPOT reader as previously described (37). Antigen-specific IFNg+ T cells were quantified by subtracting the mean IFNg SFUs in negative control wells from the mean SFUs in antigen-stimulated wells and expressed as IFNγ SFUs/10^6^ cells. A response was considered positive if it was above the cutoff value calculated from negative control wells as average mean SFU + 3 standard deviations (57 SFUs/10^6^ cells).

### Cell sorting

CD4+CD25+ and CD4+CD25− subsets were sorted by autoMACS using the CD4+CD25+ regulatory T cell isolation system (Miltenyi Biotec Inc., Auburn, CA, USA) as previously described (36). FoxP3 expression in sorted CD4+CD25+ and CD4+CD25− T cell subsets was determined by FACS to compare direct FoxP3+ Treg frequency and effector T cell suppression in subsequent *ex vivo* Treg suppression assay.

### *Ex vivo* Treg suppression assay

AutoMACS-sorted CD4+CD25+: “suppressor” T cells were cocultured in triplicate wells with autologous CD4+CD25− “responders” (60,000 cells/well) in 96-well plates at suppressor/responder ratios of 1:0, 1:1, 0.5:1, 0.25:1, 0.125:1, and 0:1 for 3 days with media alone, 2 μg/ml phytohemagglutinin (PHA; Sigma Chemical Co.), or 0.04 μg/ml anti-CD3 (clone UCHT1; BD Pharmingen) with 2 μg/ml anti-CD28 (clone CD28.2; BD Pharmingen) before 16 h of [3H]thymidine (1 μCi/well) uptake as previously described (7, 33, 36). Proliferation was expressed as a stimulation index (SI): the mean cpm in stimulated wells divided by the mean cpm in unstimulated wells. T cell proliferation in each coculture was normalized by proliferation in CD4+CD25− T cells alone and compared to the calculated percentage of FoxP3+CD4 T cells in each coculture condition, based on %FoxP3+ cells in CD4+CD25− and CD4+CD25+ cell subsets determined by FACS.

### Statistical analysis

Clinical and immunologic parameters between patient groups were compared by the non-parametric Mann–Whitney *U* test or Kruskal–Wallis. With multiple two-way comparisons, *P*-values were corrected for multiple comparisons. Pair-wise comparisons were performed by paired *t*-test. Correlations were tested for statistical significance by Spearman rank correlation. *P*-values below 0.05 were considered significant.

## Results

### FoxP3 expression is increased in CD4 T cells from HCV/HIV-coinfected than HIV or HCV-monoinfected patients

Table 1 shows the demographic, clinical, and virological characteristics of our patient groups with HCV (*n* = 20), HCV/HIV (*n* = 23), and HIV (*n* = 21) infection. They were mostly males in their 50s with preserved liver function (e.g., serum albumin, total bilirubin, INR, and platelets). Majority of HCV-infected patients had HCV genotype 1 infection, without significant differences in HCV RNA titers between HCV and HCV/HIV groups (*P* = 0.98), consistent with previous findings from our population (13). HIV viral load was undetectable in 83% in HCV/HIV-coinfected and in 61% in HIV-monoinfected patients (*P* = 0.10).

**Figure 1:**
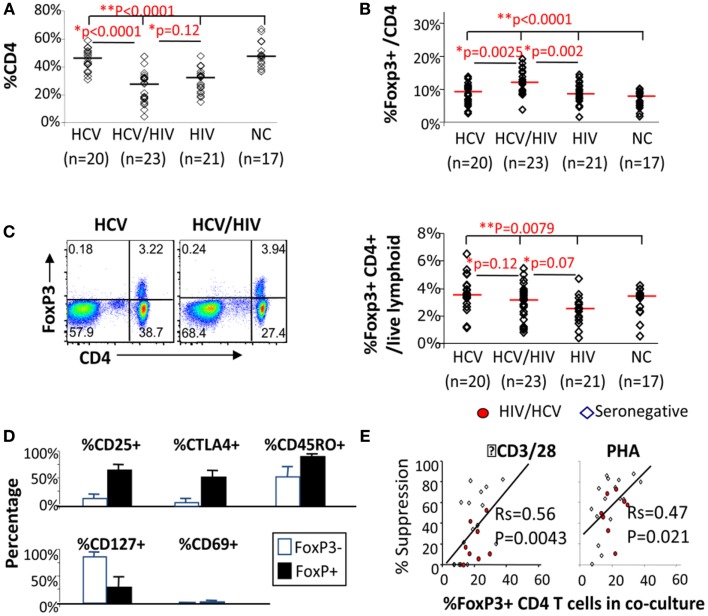
**FoxP3+ Tregs are enriched in CD4 T cells from HCV/HIV-coinfected patients**. **(A)** Frequency of CD4 T cells among viable lymphocytes is compared between 20 HCV-monoinfected (HCV), 23 HCV/HIV-coinfected (HCV/HIV), 21 HIV-monoinfected (HIV), and 17 seronegative control (NC) subjects. Horizontal bars indicate median values. *P*-values are calculated with non-parametric Kruskal–Wallis for comparison between all four groups and Mann–Whitney *U* for pair-wise comparisons. Two-way comparison of HCV/HIV coinfection group with HCV or HIV-monoinfection group was made using non-parametric Mann–Whitney *U* and corrected for multiple comparisons. **(B)** Frequency of FoxP3+CD4 T cells (FoxP3+ Tregs) in CD4 T cells is compared between 20 HCV-monoinfected (HCV), 23 HCV/HIV-coinfected (HCV/HIV), 21 HIV-monoinfected (HIV), and 17 seronegative control (NC) subjects. Horizontal bars indicate median values. *P*-values are calculated with non-parametric Kruskal–Wallis for comparison between all four groups and Mann–Whitney *U* for pair-wise comparisons. Two-way comparison of HCV/HIV coinfection group with HCV or HIV monoinfection group was made using non-parametric Mann–Whitney *U* and corrected for multiple comparisons. **(C)** *Left panel*: representative FACS plots are shown for FoxP3 and CD4 expression in live lymphocytes: *Right panel*: frequency of FoxP3+CD4+ T cells in total live lymphoid cells is compared between 20 HCV-monoinfected (HCV), 23 HCV/HIV-coinfected (HCV/HIV), 21 HIV-monoinfected (HIV), and 17 seronegative control (NC) subjects. Horizontal bars indicate median values. *P*-values are calculated with non-parametric Kruskal–Wallis for comparison between all four groups and Mann–Whitney *U* for pair-wise comparisons. Two-way comparison of HCV/HIV coinfection group with HCV or HIV monoinfection group was made using non-parametric Mann–Whitney *U* and corrected for multiple comparisons. **(D)** Phenotype of FoxP3+CD4 T cells (filled black bars) and FoxP3− CD4 T cells (unfilled bars) from HCV/HIV-coinfected patients are compared using percentage of cells expressing CD25, CTLA-4, CD45RO, CD127, and CD69. **(E)** Percent suppression of proliferation in AutoMACS-sorted CD4+CD25− “responder” T cells alone and with increasing proportion of autologous CD4+CD25+ “suppressor” T cells enriched for FoxP3+CD4 T cells upon *in vitro* stimulation with anti-CD3/CD28 or phytohemagglutinin (PHA) followed by 3H thymidine uptake. Proliferation was expressed as a stimulation index (SI): the mean cpm in stimulated wells divided by the mean cpm in unstimulated wells. T cell proliferation in each coculture was normalized by proliferation in CD4+CD25− T cells alone and compared to the calculated percentage of FoxP3+CD4 T cells in each coculture condition, based on %FoxP3+ cells in CD4+CD25− and CD4+CD25+ cell subsets determined by FACS.

We began by examining CD4 T cells for overall frequency and FoxP3 expression. As expected, CD4 T cell frequency was lower in HIV-infected subjects compared to HIV-uninfected subjects, without a significant difference between HCV/HIV-coinfected and HIV-monoinfected patients (*P* = 0.12) (Figure 1A). Notably, FoxP3 expression was enriched in CD4 T cells from HCV/HIV-coinfected patients both in frequency (HCV 8.9% vs. HCV/HIV 11.9% vs. HIV 8.5% vs. NC 7.5%, *P* < 0.0001) (Figure 1B) and mean fluorescence intensity (MFI) (Figure S1 in Supplementary Material) compared to other groups. Accordingly, FoxP3+CD4+ T cell frequency in the lymphocyte compartment was preserved in HCV/HIV-coinfected patients compared to HCV-monoinfected patients (3.2 vs. 3.5%, *P* = 0.12) despite lower CD4 T cell frequency (Figure 1C). FoxP3+CD4+ T cells from HCV/HIV-coinfected patients resembled natural Tregs with increased CD25, CTLA-4, and CD45RO expression and reduced CD127 and CD69 expression (Figure 1D) as well as dose-dependent suppression of CD4+CD25− T cell proliferation *in vitro* (Figure 1E) as previously described for HCV-monoinfected patients (36). Thus, HCV/HIV coinfection was associated with an enrichment of FoxP3+ subsets within the CD4 T cell compartment and relative preservation of FoxP3+ Tregs in circulating lymphocytes.

### T cells from HIV/HCV-coinfected patients display dysregulated expression of intrinsic T cell co-stimulatory receptors in association with FoxP3+ Treg frequency

**Figure 2:**
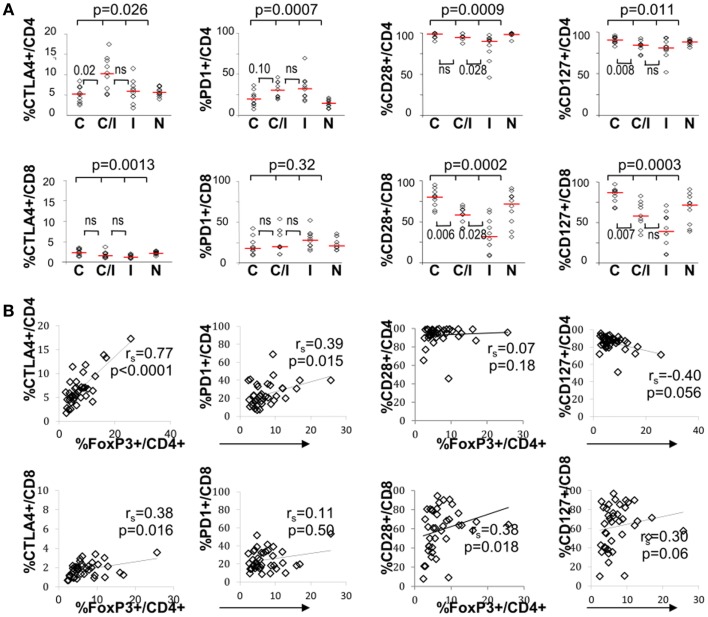
**T cell expression levels of PD-1 and/or CTLA-4 associate with FoxP3+ Tregs in patients with HCV and/or HIV infection**. **(A)** Frequency of CD4 and CD8 T cells expressing CTLA-4, PD-1, CD28, or CD127 are compared between 10 HCV-monoinfected (C), 9 HCV/HIV-coinfected (C/I), 10 HIV-monoinfected (I), and 10 seronegative control subjects (N). Horizontal bars indicate median values. *P*-values are calculated with non-parametric Kruskal–Wallis for comparison between all four groups. Two-way comparison of HCV/HIV coinfection group with HCV or HIV monoinfection group was made using non-parametric Mann–Whitney *U* and corrected for multiple comparisons. *P*-values above 0.05 before correction are shown as “ns” or “not significant.” **(B)** Frequency of CD4 and CD8 T cells expressing CTLA-4, PD-1, CD28, or CD127 are correlated with FoxP3+ Treg frequency in the CD4 T cell compartment, with non-parametric Spearman rank-order correlation and *P*-values.

The effect of differential FoxP3+ Treg enrichment on intrinsic T cell inhibitory (CTLA-4, PD-1) and co-stimulatory (CD28 and CD127) receptors was further examined in a subset of subjects with available lymphocytes. As shown in Figure 2A and Table 2, there were significant phenotypic differences in T cell subsets from the patient groups relative to HCV and/or HIV infection. First, CTLA-4 expression in CD4 T cells resembled FoxP3 in that it was highest in HCV/HIV-coinfected patients compared to other groups. Second, expression levels of PD-1, CD28, and CD127 in CD4 T cells were largely linked to HIV infection. For example, CD4 T cells in HCV/HIV-coinfected and HIV-monoinfected patients displayed PD-1 expression at twofold higher level compared to CD4 T cells from HIV-uninfected patients (Table 2). Furthermore, CD4 T cells from HCV/HIV-coinfected and HIV-monoinfected patients displayed more subtle but significantly lower CD28/CD127 expression compared to CD4 T cells from HIV-uninfected patients. Third, CTLA-4, CD28, and CD127 expression levels in CD8 T cells were significantly lower in HIV-infected than in HIV-uninfected patients. Finally, FoxP3+ Treg frequency correlated directly with CTLA-4 expression in CD4 T cells as expected (Figure 2B) but also with PD-1 expression in CD4 T cells as well as CTLA-4 and CD28 expression in CD8 T cells (Figure 2B).

**Table 2:** **Phenotype comparison of CD4 and CD8 T cells from HIV+ and HIV− subjects**.

	HIV+		HIV−		
	Median	SD	Median	SD	*P-value
**A. CD4 T CELLS**					
%PD1+	30.9	7.6	15.9	12.8	0.0001
%CTLA4+	6.5	1.6	5.3	4.1	0.06
%CD28+	93.3	3	98.8	13.1	0.0005
%CD127+	82.5	3.9	88.7	9.8	0.0024
**B. CDS T CELLS**					
0/0PDI+	26.2	9	18.5	12.8	0.112
%CTLA4+	1.2	0.6	2.2	0.7	0.0003
%CD28+	49.6	17.3	77.8	19.8	0.0001
<VoCD127+	53.9	16.8	78.8	20.8	0.0003
**C. FoxP3+CD4+ T CELLS**					
%PD1+	44	9.7	28	12.3	0.0017
%CTLA4+	53	12.6	50	10.7	0.98
%CD28+	98	0.9	99	8.9	0.06
%CD127+	25	15.2	20	11.6	0.81

*P-values by non-parametric Mann–Whitney U.

*SD, standard deviation*.

**Figure 3:**
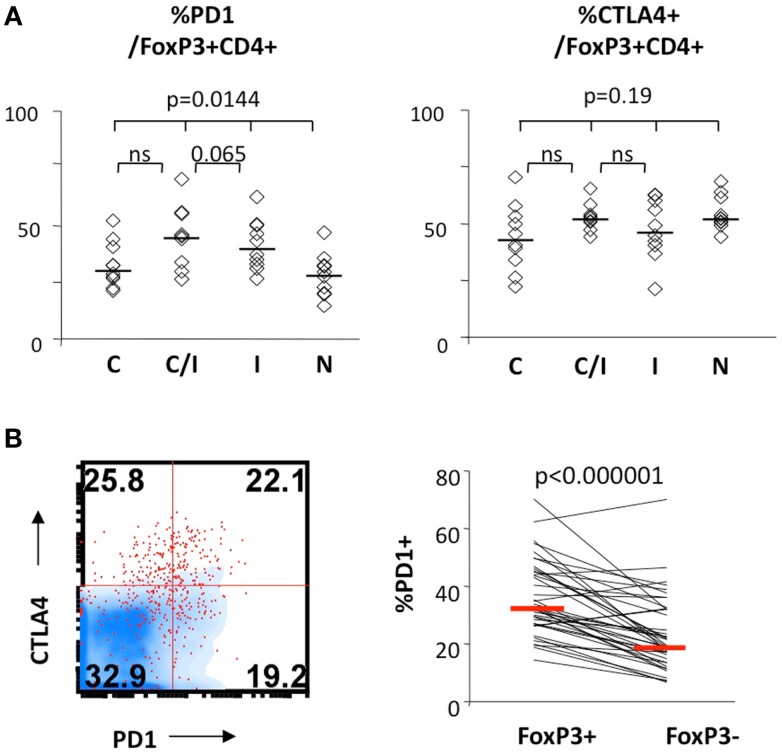
**FoxP3+ Treg expression levels of PD-1 and/or CTLA-4 in patients with HCV and/or HIV infection**. **(A)** Frequency of FoxP3+ Tregs expressing CTLA-4 and PD-1 are compared between 10 HCV-monoinfected (C), 9 HCV/HIV-coinfected (C/I), 10 HIV-monoinfected (I), and 10 seronegative control subjects (*N*). Horizontal bars indicate median values. *P*-values are calculated with non-parametric Kruskal–Wallis for comparison between all four groups. Two-way comparison of HCV/HIV coinfection group with HCV or HIV monoinfection group was made using non-parametric Mann–Whitney *U* and corrected for multiple comparisons. **(B)** Relative frequencies of cells expressing PD-1 in FoxP3+CD4 T cells and FoxP3−CD4 T cells are compared in a representative FACS overlay on the left panel (FoxP3+CD4 T cells as red dot plot, FoxP3− CD4 T cells in blue density plot) and in pair-wise comparison in 10 HCV, 9 HCV/HIV, 10 HIV, and 10 seronegative control. *P*-value is calculated by paired*t*-test.

We further examined expression of PD-1 and CTLA-4 in FoxP3+ Tregs. As shown in Figure 3, PD-1 expression differed significantly between the four patient groups, without significant differences for CTLA-4. There was no significant difference between the groups in CD28 or CD127 expression in FoxP3+ Tregs (data not shown). Differential PD-1 expression by FoxP3+ Tregs was based on HIV infection, as FoxP3+ Tregs displayed twofold higher PD-1 expression than FoxP3+ Tregs from HIV-infected patients (44 vs. 28%, *P* = 0.0017) (Table 2). Thus, HCV/HIV coinfection was associated with global dysregulation in T cell phenotype in association with FoxP3+ Treg frequency, with both HCV and HIV-mediated effects.

### FoxP3+ Treg frequency and global T cell expression of intrinsic co-stimulatory receptors correlate with CD4 T cell frequency

**Figure 4:**
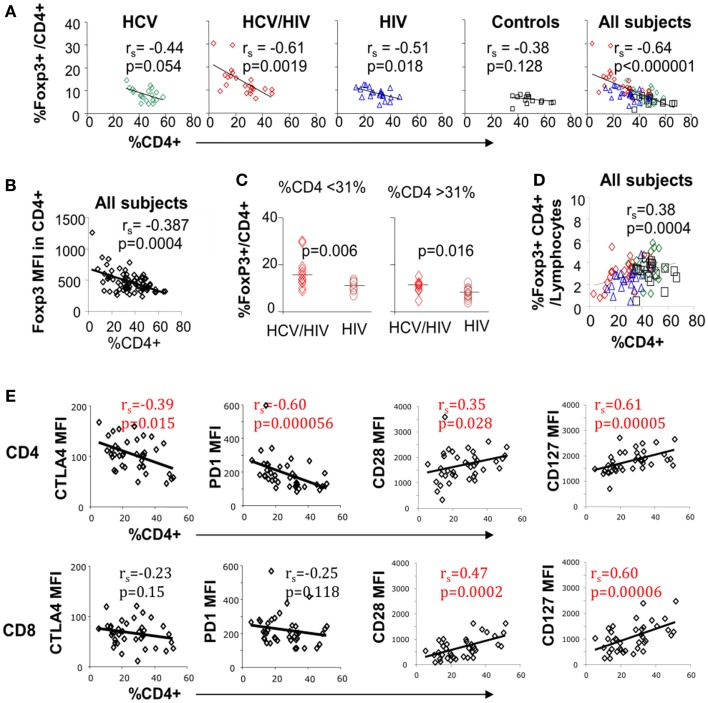
**Circulating CD4 T cell frequency is associated with FoxP3, PD-1, CTLA-4, and CD28 expression on T cell subsets**. **(A)** Frequency of FoxP3+ Tregs in CD4 T cell compartment is compared with overall CD4 T cell frequency in live lymphocytes for each patient group and with all groups combined with 20 HCV-monoinfected (HCV), 23 HCV/HIV-coinfected (HCV/HIV), 21 HIV-monoinfected (HIV), and 17 seronegative control (NC) subjects. Spearman rank-order correlation and *P*-values are shown. **(B)** FoxP3 mean fluorescence intensity (MFI) is compared with overall CD4 T cell frequency in live lymphocytes. Spearman rank-order correlation and *P*-values are shown. **(C)** Frequency of FoxP3+ Tregs in CD4 T cell compartment is compared between in HCV/HIV-coinfected and HIV-monoinfected patients with CD4 T cell frequency below or higher than a cutoff of 31% (median CD4 T cell frequency). *P*-values are calculated by non-parametric Mann–Whitney *U* test. **(D)** Frequency of FoxP3+CD4+ T cells in live lymphocyte compartment is compared with CD4 T cell frequency with Spearman rank-order correlation and *P*-values. **(E)** CTLA-4, PD-1, CD28, and CD127 mean fluorescence intensities (MFI) in CD4 and CD8 T cells are compared with overall CD4 T cell frequency in live lymphocyte compartment. Spearman rank-order correlation and *P*-values are shown.

Inverse association between FoxP3+ Treg frequency and CD4 T cell frequency was previously reported in HIV-monoinfected patients (23). In our study, inverse correlation between FoxP3+ Treg and CD4 T cell frequencies was also seen in HCV/HIV-coinfected patients as well as HIV-monoinfected patients (Figure 4A). This correlation became even stronger when results from all groups including uninfected controls were combined (Figures 4A,B far right). However, FoxP3+ Treg frequencies remained higher in HCV/HIV-coinfected than HIV-monoinfected patients in a subgroup analysis with lower or higher CD4 T cell frequencies based on a median cutoff (31%) (Figure 4C). This difference was also visually apparent by higher clustering of data points from HCV/HIV-coinfected patients on the *y*-axis (red diamonds) compared to those from HIV-monoinfected patients (blue triangles) (far right graph on Figure 4A).

Nevertheless, FoxP3+ Treg frequency in total lymphocytes decreased as overall CD4 T cell frequency declined (Figure 4D). Finally, global CTLA-4 and PD-1 expression in CD4 but not CD8 T cells correlated inversely with overall CD4 T cell frequency whereas CD28 and CD127 expression in both CD4 and CD8 T cells correlated directly with CD4 T cell frequency (Figure 4E). Thus, FoxP3+ Treg frequency was significantly associated with CD4 T cell frequency, which was further linked to T cell expression of multiple inhibitory and co-stimulatory receptors.

### FoxP3 expression in CD4 T cells is not associated with liver function parameters or level of HIV/HCV viremia

Despite its significant associations with CD4 T cell frequency and global T cell phenotype, FoxP3+ Treg frequency did not correlate with serum HCV RNA titers or liver function parameters (Figure S2A in Supplementary Material). Similarly, T cell expression of PD-1, CTLA-4, or CD28 did not correlate with HCV RNA titers or clinical liver function parameters (data not shown). As previously reported (38–41), age was positively associated with FoxP3+ Treg frequency (Figure S2B in Supplementary Material) but not with CD4 T cell frequency or co-stimulatory receptor expression on CD4 and CD8 T cells (data not shown). We also did not find an association between HIV viral load and FoxP3+ Treg frequency. For example, FoxP3+ Treg frequency did not differ between HIV-seropositive patients with and without active HIV viremia (Figure S2C in Supplementary Material, left panel). Furthermore, FoxP3+ Treg frequency did not correlate with HIV viral load among actively HIV-viremic patients (Figure S2C in Supplementary Material, middle panel), and it remained higher in HCV/HIV-coinfected patients with undetectable HIV viremia compared to HIV-monoinfected patients with undetectable HIV viremia and HCV-monoinfected patients (Figure S2C in Supplementary Material, right panel).

### Virus-specific effector T cell responses in HCV/HIV-coinfected patients do not correlate with FoxP3+ Treg frequency or immune inhibitory receptor expression levels on T cells

**Figure 5:**
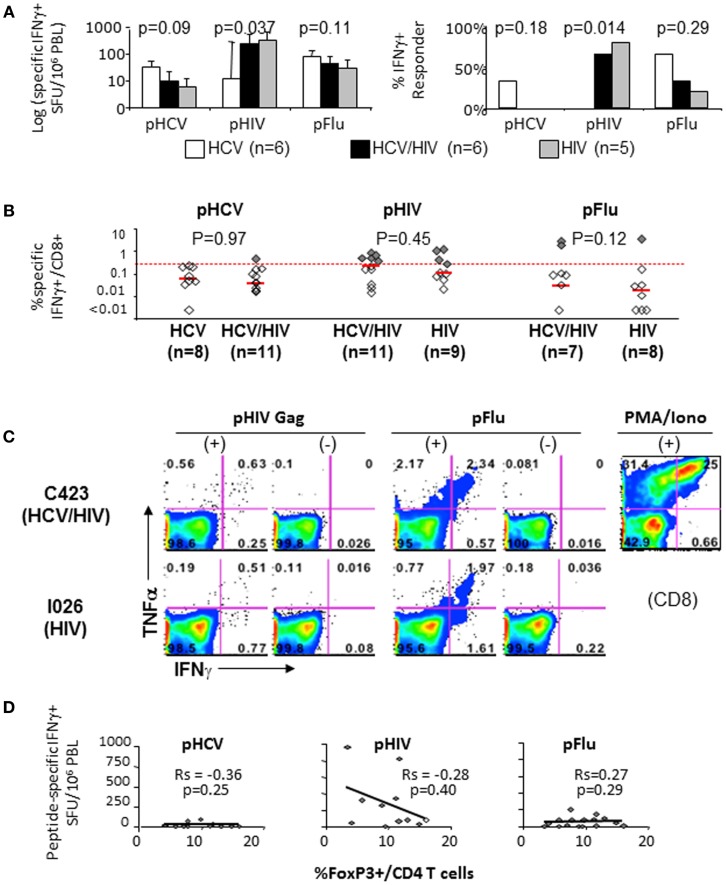
**T cell cytokine responses to HCV, HIV, and Flu peptides**. **(A)** Median T cell IFNγ responses and percent positive responders to HCV, HIV, and Flu peptides in HCV, HCV/HIV, and HIV-infected patient groups are shown in log scale (log spot forming units per million PBMC) based on IFNγ ELISPOT assay using PBMCs stimulated *ex vivo* with overlapping 15mer peptide pools spanning HCV NS3 (pHCV), HIV Gag (pHIV), and influenza matrix (pFlu). Unfilled bar (HCV-monoinfected, *n* = 6), black bar (HCV/HIV-coinfected, *n* = 6), gray bar (HIV-monoinfected, *n* = 5). Positive responses were defined by cutoff values for each assay (>average background + 3 SDs). *P*-values comparing three groups were calculated by non-parametric Kruskal–Wallis with only significant difference seen for HIV-specific T cell response (both by SFU and % responders) due to lack of HIV-specific T cell response in HCV-monoinfected patients. **(B)** Frequency of CD8+ T cells with antigen-specific IFNγ production in short term PBMC cultures is shown from HCV/HIV-coinfected patients, HCV-monoinfected, and HIV-monoinfected patients. PBMC cultures were stimulated for 7 days with overlapping peptide pools (2 μM) and rIL-2 followed by further 5.5 h peptide stimulation in the presence of Brefeldin A, intracellular cytokine staining, and FACS analysis. Total of 8 HCV/HIV-coinfected patients, 11 HCV-monoinfected, and 9 HIV-monoinfected patients were included, with sample size for each stimulation condition shown as defined by available lymphocytes. Red dotted line indicates the 0.27%, which was the cutoff value for a positive response based on average background + 3 SDs. Positive responses above 0.27% are further highlighted as dark shaded diamonds. *P*-values comparing two groups were calculated by non-parametric Mann–Whitney *U* without significant differences between the groups. **(C)** Representative FACS plots show TNFα and IFNγ expression in CD8-gated cells from an HCV/HIV-coinfected (C423) and HIV-monoinfected (I026) subjects. **(D)** Peptide-specific IFNγ response (in spot forming units per million PBMC) is compared with the frequency of FoxP3+ Tregs in CD4 T cell compartment from HCV/HIV-coinfected patients. Spearman rank-order correlation and *P*-values are shown without significant associations.

Effector T cell responses to HCV NS3, HIV Gag, and influenza matrix peptides were compared between the patient groups with IFNγ Elispot *ex vivo* (Figure 5A) and with short term *in vitro* culture followed by intracellular cytokine staining (Figures 5B,C). In general, T cell response to HCV was weak in HCV-infected patients, whereas T cell response to HIV was more readily detected in HIV-infected patients (Figures 5A,B). In particular, HCV/HIV-coinfected patients displayed weak to undetectable T cell IFNγ response to HCV that contrasted with HIV-specific T cell IFNγ responses that were readily detected at levels similar to HIV-monoinfected patients (Figures 5A–C). Relevant for immune regulatory pathways, T cell IFNγ responses to HCV, HIV, or influenza peptides did not correlate with FoxP3+ Treg frequency (Figure 5D) or T cell expression of PD-1 or CTLA-4 (data not shown).

**Figure 6:**
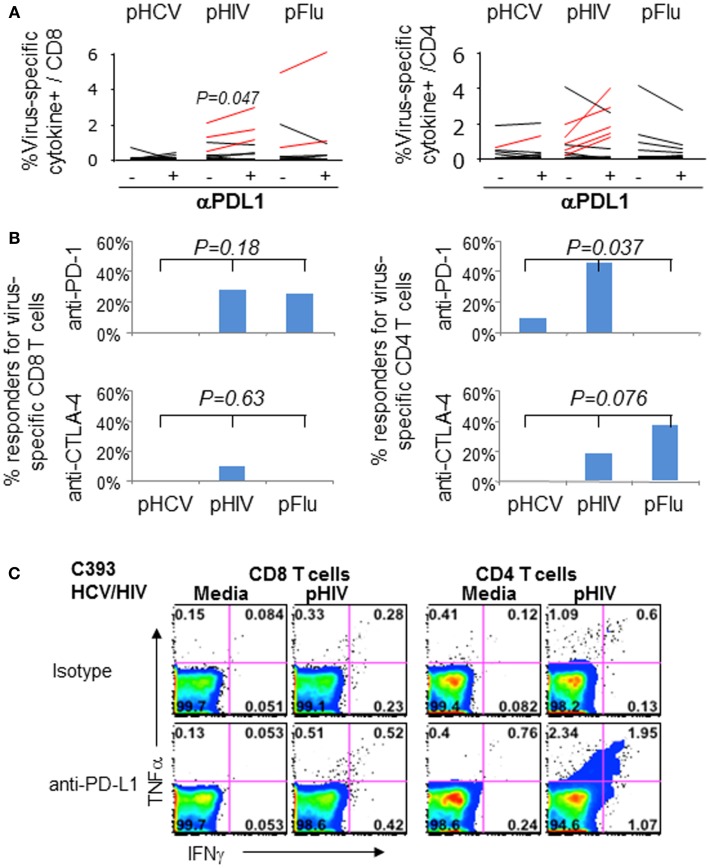
**Effect of *in vitro* PD-1 blockade on virus-specific T cell cytokine responses in HCV/HIV-coinfected patients**. T cell cytokine responses (combining IFNγ and/or TNFα responses) to HCV, HIV, and Flu peptides were examined in HCV/HIV-coinfected patients with and without PD-1 and/or CTLA-4 blockade *in vitro*. PBMCs from HCV/HIV-coinfected patients were cultured for 7 days *in vitro* with pHCV NS3, pHIV GAG, or pFlu Matrix in the presence of isotype control or blocking antibodies. The 7-day cultures were further stimulated for 6 h with media alone or with each peptide before intracellular IFN-γ and TNF-α staining. Virus-specific responses were calculated by subtracting the cytokine responses in media control samples from peptide-stimulated samples during the intracellular cytokine staining. A positive response to blockade was defined as antigen-specific response that was greater than the isotype control condition by at least a standard deviation of the all background responses (0.36%). **(A)** Percentage of virus-specific cytokine+ cells in CD8 (left panel) and CD4 T cells (right panel) are shown from HCV/HIV-coinfected patients, comparing responses with isotype antibody and with anti-PDL1 blocking Ab (10 μg/ml). Significant *P*-value per paired *t*-test is shown for HIV Gag. Assay pairs with augmented responses to PD-1 blockade above isotype control condition are highlighted as red lines. Significant increase in cytokine response by PD-1 blockade is shown for HIV-specific CD8 T cells (*P* = 0.047) via paired *t*-test. **(B)** Percentage of positive responders (augmentations above 0.36%) to anti-PDL1 or anti-CTLA-4 is shown for virus-specific CD8 (left panel) and CD4 T cells (right panel) from HCV/HIV-coinfected patients. Significant anti-PDL1-mediated augmentation is shown for HIV-specific CD4 T cell response by Kruskal–Wallis (*P* = 0.037). **(C)** Representative FACS plots are shown for HIV Gag-specific IFN-γ and TNF-α production in CD8 (left) and CD4 T cells (right) in short term PBMC culture from HCV/HIV-coinfected patients, following 7 day culture with HIV peptides in the presence of either isotype antibody or anti-PDL1. Marked induction in IFNγ and TNFα responses is apparent for CD4 T cells following PD-1 blockade.

We then examined if blockade of PD-1 and/or CTLA-4 with anti-PDL1 and anti-CTLA-4 could enhance virus-specific effector T cell responses in 11 HCV/HIV-coinfected patients. As shown in Figures 6A–C, anti-PD-1 augmented HIV-specific CD8 T cell response in 3/11 patients and HIV-specific CD4 T cell response in 5/11 patients. By contrast, PD-1 blockade augmented HCV-specific CD8 T cell responses in 0/11 patients and HCV-specific CD4 T cell response in 1/11 patients. This resulted in anti-PDL1-mediated augmentations for 8/22 HIV-specific T cell responses and 1/22 HCV-specific T cell response (36 vs. 4.5%, *P* = 0.02). As for CTLA-4 blockade, there was no augmentation for HCV-specific T cell responses compared to 3/22 HIV-specific T cell responses (0 vs. 14%, *P* = NS). Augmentations were also observed for Flu-specific CD4 T cell response upon CTLA-4 but not PD-1 blockade. Combined PD-1/CTLA-4 blockade did not further enhance antiviral T cell function (data not shown), consistent with our previous study regarding peripheral blood compartment (6, 7).

Collectively, we show that multiple immune regulatory pathways are induced in HCV/HIV coinfection including FoxP+ Tregs, PD-1, and CTLA-4 in significant association with HIV-associated CD4 T cell loss and with apparent suppression of effector T cell responses against HIV but not HCV.

## Discussion

Effector functions of virus-specific CD8 T cells are regulated by extrinsic immune regulatory pathways including Tregs and cytokines as well as intrinsic expression of immune modulatory molecules (9, 42–45). In viral infections, these regulatory pathways can limit the extent of cellular injury but also promote viral survival with long-term pathogenetic consequences. In particular, HCV persists with functionally impaired HCV-specific effector T cells that over-express PD-1, CTLA-4, and/or Tim-3 (6, 7, 45–47) and increased circulating FoxP3+ Tregs that are indistinguishable from natural Tregs (33, 36, 48, 49). Treg development and function may in turn be influenced by PD-1 and CD28 (17, 50). Further complexity is expected in HCV/HIV coinfection due to HIV-mediated immune effects as well as Treg dynamics (23, 51). For example, PD-1 and CTLA-4 expression in antiviral T cells correlated with HIV viral load and CD4 T cell count in HIV-monoinfected patients (3–5). With HCV/HIV coinfection, Treg induction has been related to HIV rather than HCV (24, 30) whereas PD-1/Tim-3 co-expression in both total and HCV-specific T cells associated with liver-related outcomes (52). In this study, the interplay between Tregs and intrinsic co-stimulatory pathways in HCV/HIV coinfection was examined with the hypothesis that HCV/HIV coinfection will heighten immune dysregulation associated with chronic viral infection.

In fact, FoxP3+ Treg frequency in total lymphocytes was similar between HCV/HIV-coinfected and HCV-monoinfected patients despite almost twofold difference in CD4 T cell frequency between HCV/HIV-coinfected and HCV-monoinfected patients. This was due to significantly enriched FoxP3 expression in CD4 T cells from HCV/HIV-coinfected patients compared to other groups. Increased FoxP3 expression in CD4 T cells from HCV/HIV-coinfected patients was not due to active HIV viremia since most patients (83%) had HIV viral load below detection limit. FoxP3+ Tregs from HCV/HIV-coinfected patients generally resembled natural Tregs in phenotype and suppressive function (Figure 1). However, FoxP3+ Tregs from HCV/HIV-coinfected patients (as well as HIV-monoinfected patients) displayed increased PD-1 expression compared to Tregs from HIV-uninfected subjects, suggesting that HIV-mediated immune activation extends to FoxP3+ Tregs.

Perhaps due to the preserved FoxP3+ Treg frequency in total lymphocytes, there was no significant association between FoxP3+ Treg frequency and HCV-associated liver disease parameters or HCV RNA titers. However, FoxP3 expression in CD4 T cells was inversely correlated with overall CD4 T cell frequency, a critical marker of immune competence. While this inverse association was previously reported in HIV-monoinfected patients (23), our study further extends this association to HCV/HIV-coinfected patients. Perhaps more interestingly, the inverse association between CD4 T cell frequency and FoxP3+ Treg frequency became even stronger when all subjects (including the uninfected controls) were included (rs = −0.64, *P* < 0.000001). This suggested a more generalized homeostatic mechanism to sustain FoxP3+ Treg frequency as CD4 T cell frequency declines. This possibility was previously reported for cancer patients undergoing chemotherapy (53), but may be more generalized based on our findings. In this regard, HIV-mediated CD4 T cell loss may play a dominant effect on FoxP3+ Treg dynamics to preserve the total circulating FoxP3+ Tregs (24, 30), although this is insufficient with progressive HIV-associated CD4 T cell loss.

The main finding in our study is that multiple immune inhibitory pathways are induced HCV/HIV-coinfected patients due to HCV and/or HIV infection, despite therapeutic HIV suppression. At the same time, both CD4 and CD8 T cells in HCV/HIV-coinfected patients showed reduced expression of positive co-stimulatory receptor CD28 and IL7 receptor alpha CD127. FoxP3+ Treg frequency correlated with expression of PD-1 and CTLA-4 on CD4 T cells as well as CTLA-4 and CD28 expression on CD8 T cells. These associations are likely linked to CD4 T cell frequency in which CD4 T cell loss occurs with preferential induction of multiple inhibitory pathways in CD4 (but not CD8) T cells and reduction in CD28 and CD127 expression for both CD4 and CD8 T cells. It is not clear if these changes are the cause or consequence of CD4 T cell loss. Furthermore, immune inhibitory induction despite apparent therapeutic HIV suppression suggests that virus control is not absolute and that there is ongoing HIV-associated immune activation.

Blockade of one or more co-stimulatory receptor including PD-1, CTLA-4, Tim-3, and 4-1BB has been shown to enhance virus-specific T cell function in chronic viral infections including HCV, HBV, HIV, and SIV (2–12, 31). Better understanding of these pathways has therapeutic implications, given the available agents to modulate these pathways to treat chronic viral infection and enhance tumor immunity. For example, PD-1 blockade *in vivo* improved control of SIV *in vivo* with enhanced gut immunity, reduced microbial translocation, and survival of SIV-infected rhesus macaques (11, 12). Furthermore, PD-1 blockade *in vivo* was well-tolerated and promoted virus control in a subset of HCV-infected patients (54) whereas CTLA-4 blockade in patients with hepatocellular carcinoma and chronic hepatitis C was tolerated with antiviral and immune stimulatory effects (32). Our findings in HCV/HIV-coinfected patients show that co-stimulatory receptor blockade was more effective for T cell responses to HIV than HCV. This may in part reflect the underlying frequency as well as functionality of HCV-specific T cells. Since most HCV/HIV-coinfected patients in our study had therapeutic suppression of HIV (but not HCV), HIV-specific T cells may have been less exhausted and paradoxically more responsive to co-stimulatory blockade than HCV-specific T cells. In this regard, we previously showed that PD-1 blockade was less effective for highly exhausted HCV-specific T cells isolated from HCV-infected liver (6, 7). Alternatively, HCV-specific effector T cells may be poorly maintained in HCV/HIV-coinfected patients due to HIV-associated CD4 T cell dysfunction and immune inhibition. It is also possible that HCV and HIV reside in different compartments although we previously showed that *in vitro* PD-1 blockade of PBMC can enhance HCV-specific effector T cell function in four/six HCV-infected patients (6).

Collectively, we show that multiple T cell inhibitory pathways are induced in patients with HCV/HIV coinfection. These changes were inversely associated with circulating CD4 T cell frequencies as a consequence of HIV infection, but not with viral titers or HCV-associated liver function parameters. *In vitro* PD-1 blockade of PBMC from HCV/HIV-coinfected patients restored effector T cell responses to HIV but not HCV with less prominent impact by CTLA-4 blockade. These findings provide new insights on interplay between extrinsic and intrinsic immune regulatory pathways as well as virus-specific effect of co-stimulatory receptor blockade relevant for immunotherapeutic development.

## Conflict of Interest Statement

There is no financial conflict of interest. However, Kyong-Mi Chang has family member employed by Bristol Myers Squibb, which makes biologics related to immune inhibitory blockade.

## Supplementary Material

The Supplementary Material for this article can be found online at http://www.frontiersin.org/Journal/10.3389/fimmu.2014.00265/abstract

## References

[1] Barber DL Wherry EJ Masopust D Zhu B Allison JP Sharpe AH Restoring function in exhausted CD8 T cells during chronic viral infection. Nature (2006) 439:682–7 10.1038/nature04444 16382236

[2] Freeman GJ Wherry EJ Ahmed R Sharpe AH Reinvigorating exhausted HIV-specific T cells via PD-1-PD-1 ligand blockade. J Exp Med (2006) 203:2223–7 10.1084/jem.20061800 17000870 PMC2118103

[3] Day CL Kaufmann DE Kiepiela P Brown JA Moodley ES Reddy S PD-1 expression on HIV-specific T cells is associated with T-cell exhaustion and disease progression. Nature (2006) 443:350–4 10.1038/nature05115 16921384

[4] Kaufmann DE Walker BD Programmed death-1 as a factor in immune exhaustion and activation in HIV infection. Curr Opin HIV AIDS (2008) 3:362–7 10.1097/COH.0b013e3282f9ae8b 19372991

[5] Kaufmann DE Kavanagh DG Pereyra F Zaunders JJ Mackey EW Miura T Upregulation of CTLA-4 by HIV-specific CD4(+) T cells correlates with disease progression and defines a reversible immune dysfunction. Nat Immunol (2007) 8:1246–54 10.1038/ni1515 17906628

[6] Nakamoto N Kaplan DE Coleclough J Li Y Valiga ME Kaminski M Functional restoration of HCV-specific CD8 T cells by PD-1 blockade is defined by PD-1 expression and compartmentalization. Gastroenterology (2008) 134:1937.e1–2 10.1053/j.gastro.2008.02.033 18549878 PMC2665722

[7] Nakamoto N Cho H Shaked A Olthoff K Valiga ME Kaminski M Synergistic reversal of intrahepatic HCV-specific CD8 T cell exhaustion by combined PD-1/CTLA-4 blockade. PLoS Pathog (2009) 5:e1000313 10.1371/journal.ppat.1000313 19247441 PMC2642724

[8] Tzeng HT Tsai HF Liao HJ Lin YJ Chen L Chen PJ PD-1 blockage reverses immune dysfunction and hepatitis B viral persistence in a mouse animal model. PLoS One (2012) 7:e39179 10.1371/journal.pone.0039179 22761734 PMC3382233

[9] Fisicaro P Valdatta C Massari M Loggi E Ravanetti L Urbani S Combined blockade of programmed death-1 and activation of CD137 increase responses of human liver T cells against HBV, but not HCV. Gastroenterology (2012) 143(6):1576–85.e4 10.1053/j.gastro.2012.08.041 22929808

[10] Boni C Fisicaro P Valdatta C Amadei B Di Vincenzo P Giuberti T Characterization of hepatitis B virus (HBV)-specific T-cell dysfunction in chronic HBV infection. J Virol (2007) 81:4215–25 10.1128/JVI.02844-06 17287266 PMC1866111

[11] Dyavar Shetty R Velu V Titanji K Bosinger SE Freeman GJ Silvestri G PD-1 blockade during chronic SIV infection reduces hyperimmune activation and microbial translocation in rhesus macaques. J Clin Invest (2012) 122:1712–6 10.1172/JCI60612 22523065 PMC3336983

[12] Estes JD Enhancing immune responses to limit chronic immune activation during SIV. J Clin Invest (2012) 122:1611–4 10.1172/JCI63389 22523060 PMC3337001

[13] Anderson KB Guest JL Rimland D Hepatitis C virus coinfection increases mortality in HIV-infected patients in the highly active antiretroviral therapy era: data from the HIV Atlanta VA Cohort Study. Clin Infect Dis (2004) 39:1507–13 10.1086/425360 15546088

[14] Merriman NA Porter SB Brensinger CM Reddy KR Chang KM Racial difference in mortality among U.S. veterans with HCV/HIV coinfection. Am J Gastroenterol (2006) 101(4):760–7 10.1111/j.1572-0241.2006.00531.x 16494582

[15] Wan YY Regulatory T cells: immune suppression and beyond. Cell Mol Immunol (2010) 7:204–10 10.1038/cmi.2010.20 20383175 PMC2868372

[16] Fife BT Bluestone JA Control of peripheral T-cell tolerance and autoimmunity via the CTLA-4 and PD-1 pathways. Immunol Rev (2008) 224:166–82 10.1111/j.1600-065X.2008.00662.x 18759926

[17] Zhang R Huynh A Whitcher G Chang J Maltzman JS Turka LA An obligate cell-intrinsic function for CD28 in Tregs. J Clin Invest (2013) 123:580–93 10.1172/JCI65013 23281398 PMC3561819

[18] Sage PT Francisco LM Carman CV Sharpe AH The receptor PD-1 controls follicular regulatory T cells in the lymph nodes and blood. Nat Immunol (2013) 14:152–61 10.1038/ni.2496 23242415 PMC3788614

[19] Kaufmann DE Walker BD PD-1 and CTLA-4 inhibitory cosignaling pathways in HIV infection and the potential for therapeutic intervention. J Immunol (2009) 182:5891–7 10.4049/jimmunol.0803771 19414738 PMC3726306

[20] Elrefaei M Baker CA Jones NG Bangsberg DR Cao H Presence of suppressor HIV-specific CD8+ T cells is associated with increased PD-1 expression on effector CD8+ T cells. J Immunol (2008) 180:7757–63 10.4049/jimmunol.180.11.7757 18490780 PMC3591725

[21] Bi X Suzuki Y Gatanaga H Oka S High frequency and proliferation of CD4+ FOXP3+ Treg in HIV-1-infected patients with low CD4 counts. Eur J Immunol (2009) 39:301–9 10.1002/eji.200838667 19089812

[22] Cao W Jamieson BD Hultin LE Hultin PM Detels R Regulatory T cell expansion and immune activation during untreated HIV type 1 infection are associated with disease progression. AIDS Res Hum Retroviruses (2009) 25:183–91 10.1089/aid.2008.0140 19239357 PMC2782619

[23] Schulze Zur Wiesch J Thomssen A Hartjen P Toth I Lehmann C Meyer-Olson D Comprehensive analysis of frequency and phenotype of T regulatory cells in HIV infection: CD39 expression of FoxP3+ T regulatory cells correlates with progressive disease. J Virol (2011) 85:1287–97 10.1128/JVI.01758-10 21047964 PMC3020516

[24] Rallon NI Lopez M Soriano V Garcia-Samaniego J Romero M Labarga P Level, phenotype and activation status of CD4+FoxP3+ regulatory T cells in patients chronically infected with human immunodeficiency virus and/or hepatitis C virus. Clin Exp Immunol (2009) 155:35–43 10.1111/j.1365-2249.2008.03797.x 19076827 PMC2665677

[25] Suchard MS Mayne E Green VA Shalekoff S Donninger SL Stevens WS FOXP3 expression is upregulated in CD4T cells in progressive HIV-1 infection and is a marker of disease severity. PLoS One (2010) 5:e11762 10.1371/journal.pone.0011762 20668701 PMC2909259

[26] Prendergast A Prado JG Kang YH Chen F Riddell LA Luzzi G HIV-1 infection is characterized by profound depletion of CD161+ Th17 cells and gradual decline in regulatory T cells. AIDS (2010) 24:491–502 10.1097/QAD.0b013e3283344895 20071976

[27] Apoil PA Puissant B Roubinet F Abbal M Massip P Blancher A FOXP3 mRNA levels are decreased in peripheral blood CD4+ lymphocytes from HIV-positive patients. J Acquir Immune Defic Syndr (2005) 39:381–5 10.1097/01.qai.0000169662.30783.2d 16010156

[28] Sachdeva M Fischl MA Pahwa R Sachdeva N Pahwa S Immune exhaustion occurs concomitantly with immune activation and decrease in regulatory T cells in viremic chronically HIV-1-infected patients. J Acquir Immune Defic Syndr (2010) 54:447–54 10.1097/QAI.0b013e3181e0c7d0 20463584 PMC3095513

[29] Epple HJ Loddenkemper C Kunkel D Troger H Maul J Moos V Mucosal but not peripheral FOXP3+ regulatory T cells are highly increased in untreated HIV infection and normalize after suppressive HAART. Blood (2006) 108:3072–8 10.1182/blood-2006-04-016923 16728694

[30] Zhuang Y Wei X Li Y Zhao K Zhang J Kang W HCV coinfection does not alter the frequency of regulatory T cells or CD8+ T cell immune activation in chronically infected HIV+ Chinese subjects. AIDS Res Hum Retroviruses (2012) 28:1044–51 10.1089/AID.2011.0318 22214236

[31] Schurich A Khanna P Lopes AR Han KJ Peppa D Micco L Role of the coinhibitory receptor cytotoxic T lymphocyte antigen-4 on apoptosis-Prone CD8 T cells in persistent hepatitis B virus infection. Hepatology (2011) 53:1494–503 10.1002/hep.24249 21360567

[32] Sangro B Gomez-Martin C de la Mata M Inarrairaegui M Garralda E Barrera P A clinical trial of CTLA-4 blockade with tremelimumab in patients with hepatocellular carcinoma and chronic hepatitis C. J Hepatol (2013) 59:81–8 10.1016/j.jhep.2013.02.022 23466307

[33] Sugimoto K Ikeda F Stadanlick J Nunes FA Alter HJ Chang KM Suppression of HCV-specific T cells without differential hierarchy demonstrated *ex vivo* in persistent HCV infection. Hepatology (2003) 38:1437–48 10.1053/jhep.2003.09026 14647055

[34] Sugimoto K Kaplan DE Ikeda F Ding J Schwartz J Nunes FA Strain-specific T-cell suppression and protective immunity in patients with chronic hepatitis C virus infection. J Virol (2005) 79:6976–83 10.1128/JVI.79.11.6976-6983.2005 15890937 PMC1112102

[35] Ferre AL Lemongello D Hunt PW Morris MM Garcia JC Pollard RB Immunodominant HIV-specific CD8+ T-cell responses are common to blood and gastrointestinal mucosa, and Gag-specific responses dominate in rectal mucosa of HIV controllers. J Virol (2010) 84:10354–65 10.1128/JVI.00803-10 20668079 PMC2937770

[36] Ebinuma H Nakamoto N Li Y Price DA Gostick E Levine BL Identification and *in vitro* expansion of functional antigen-specific CD25+ FoxP3+ regulatory T cells in hepatitis C virus infection. J Virol (2008) 82:5043–53 10.1128/JVI.01548-07 18337568 PMC2346728

[37] Kaplan DE Sugimoto K Newton K Valiga ME Ikeda F Aytaman A Discordant role of CD4 T-cell response relative to neutralizing antibody and CD8 T-cell responses in acute hepatitis C. Gastroenterology (2007) 132:654–66 10.1053/j.gastro.2006.11.044 17258733

[38] Gregg R Smith CM Clark FJ Dunnion D Khan N Chakraverty R The number of human peripheral blood CD4+ CD25high regulatory T cells increases with age. Clin Exp Immunol (2005) 140:540–6 10.1111/j.1365-2249.2005.02798.x 15932517 PMC1809384

[39] Rosenkranz D Weyer S Tolosa E Gaenslen A Berg D Leyhe T Higher frequency of regulatory T cells in the elderly and increased suppressive activity in neurodegeneration. J Neuroimmunol (2007) 188:117–27 10.1016/j.jneuroim.2007.05.011 17582512

[40] Lages CS Suffia I Velilla PA Huang B Warshaw G Hildeman DA Functional regulatory T cells accumulate in aged hosts and promote chronic infectious disease reactivation. J Immunol (2008) 181:1835–48 10.4049/jimmunol.181.3.1835 18641321 PMC2587319

[41] Jagger A Shimojima Y Goronzy JJ Weyand CM Regulatory T cells and the immune aging process: a mini-review. Gerontology (2014) 60(2):130–7 10.1159/000355303 24296590 PMC4878402

[42] Wherry EJ T cell exhaustion. Nat Immunol (2011) 12:492–9 21739672 10.1038/ni.2035

[43] Chevalier MF Weiss L The split personality of regulatory T cells in HIV infection. Blood (2013) 121(1):29–37 10.1182/blood-2012-07-409755 23043072

[44] Virgin HW Wherry EJ Ahmed R Redefining chronic viral infection. Cell (2009) 138:30–50 10.1016/j.cell.2009.06.036 19596234

[45] Golden-Mason L Palmer BE Kassam N Townshend-Bulson L Livingston S McMahon BJ Negative immune regulator Tim-3 is overexpressed on T cells in hepatitis C virus infection and its blockade rescues dysfunctional CD4+ and CD8+ T cells. J Virol (2009) 83:9122–30 10.1128/JVI.00639-09 19587053 PMC2738247

[46] Radziewicz H Ibegbu CC Fernandez ML Workowski KA Obideen K Wehbi M Liver-infiltrating lymphocytes in chronic human hepatitis C virus infection display an exhausted phenotype with high levels of PD-1 and low levels of CD127 expression. J Virol (2007) 81:2545–53 10.1128/JVI.02021-06 17182670 PMC1865979

[47] McMahan RH Golden-Mason L Nishimura MI McMahon BJ Kemper M Allen TM Tim-3 expression on PD-1+ HCV-specific human CTLs is associated with viral persistence, and its blockade restores hepatocyte-directed *in vitro* cytotoxicity. J Clin Invest (2010) 120:4546–57 10.1172/JCI43127 21084749 PMC2994339

[48] Boettler T Spangenberg HC Neumann-Haefelin C Panther E Urbani S Ferrari C T cells with a CD4+CD25+ regulatory phenotype suppress *in vitro* proliferation of virus-specific CD8+ T cells during chronic hepatitis C virus infection. J Virol (2005) 79:7860–7 10.1128/JVI.79.12.7860-7867.2005 15919940 PMC1143651

[49] Cabrera R Tu Z Xu Y Firpi RJ Rosen HR Liu C An immunomodulatory role for CD4(+)CD25(+) regulatory T lymphocytes in hepatitis C virus infection. Hepatology (2004) 40:1062–71 10.1002/hep.20454 15486925

[50] Franceschini D Paroli M Francavilla V Videtta M Morrone S Labbadia G PD-L1 negatively regulates CD4+CD25+Foxp3+ Tregs by limiting STAT-5 phosphorylation in patients chronically infected with HCV. J Clin Invest (2009) 119:551–64 10.1172/JCI36604 19229109 PMC2648671

[51] Imamichi H Lane HC Regulatory T cells in HIV-1 infection: the good, the bad, and the ugly. J Infect Dis (2012) 205:1479–82 10.1093/infdis/jis238 22457283

[52] Vali B Jones RB Sakhdari A Sheth PM Clayton K Yue FY HCV-specific T cells in HCV/HIV co-infection show elevated frequencies of dual Tim-3/PD-1 expression that correlate with liver disease progression. Eur J Immunol (2010) 40:2493–505 10.1002/eji.201040340 20623550

[53] Rech AJ Mick R Kaplan DE Chang KM Domchek SM Vonderheide RH Homeostasis of peripheral FoxP3(+) CD4 (+) regulatory T cells in patients with early and late stage breast cancer. Cancer Immunol Immunother (2010) 59:599–607 10.1007/s00262-009-0780-x 19855964 PMC11030825

[54] Gardiner D Lalezari J Lawitz E DiMicco M Ghalib R Reddy KR A randomized, double-blind, placebo-controlled assessment of BMS-936558, a fully human monoclonal antibody to programmed death-1 (PD-1), in patients with chronic hepatitis C virus infection. PLoS One (2013) 8:e63818 10.1371/journal.pone.0063818 23717490 PMC3661719

