# Supplementary material for: Induction of Multiple Immune Regulatory Pathways with Differential Impact in HCV/HIV Coinfection
Source: Front Immunol. 2014 Jul 8;5:265. doi: 10.3389/fimmu.2014.00265 (PMC4086204; doi:10.3389/fimmu.2014.00265)
Supplement: Supplementary file 1 [file Presentation_1.PDF]

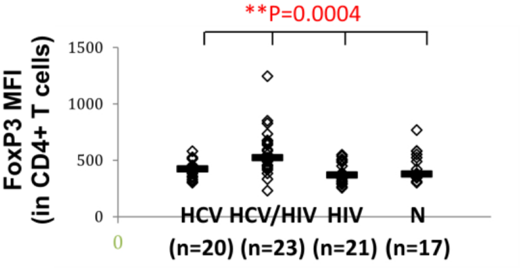

Supplementary Figure S1. FoxP3 MFI in CD4 T cells compared between 4 patient groups. P-value was calculated by Kruskal Wallis.
