# Supplementary material for: Induction of Multiple Immune Regulatory Pathways with Differential Impact in HCV/HIV Coinfection
Source: Front Immunol. 2014 Jul 8;5:265. doi: 10.3389/fimmu.2014.00265 (PMC4086204; doi:10.3389/fimmu.2014.00265)
Supplement: Supplementary file 2 [file Presentation_2.PDF]

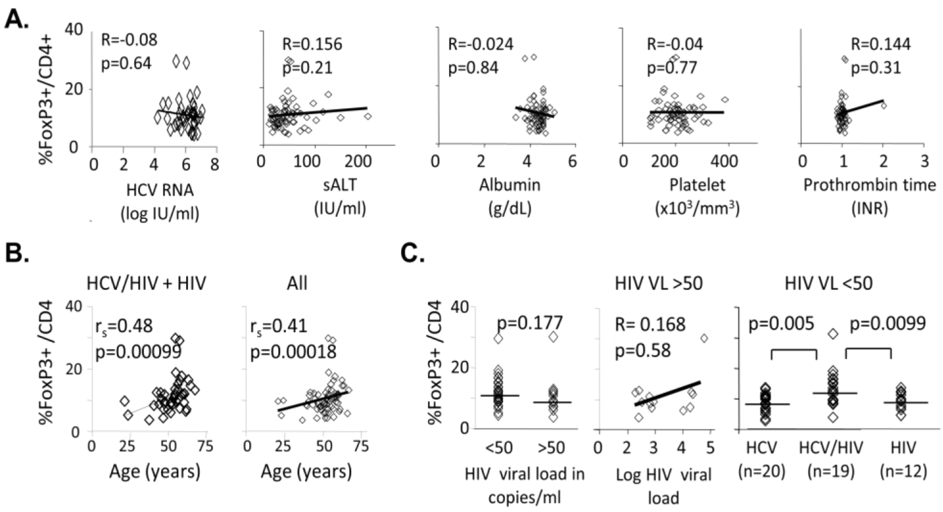

Supplementary Figure S2. Treg frequency does not correlate with HIV or HCV viral load, duration of HIV infection, or liver function parameters. (A) Frequency of FoxP3+ Tregs in CD4 T cell compartment is compared with HCV viral load (log IU/ml) or liver function parameters including serum alanine aminotransferase (sALT), serum albumin, platelet count and prothrombin time (INR) among HIV and/or HCV-infected patients. No significant correlations are observed. **(B)** Frequency of FoxP3+ Tregs in CD4 T cell compartment is compared with age among HCV/HIV and HIV-infected patients (left panel) and in all subjects combined (right panel) with significant positive correlations seen for both by Spearman Rank-Order Correlation. **(C)** Frequency of FoxP3+ Tregs in CD4 T cell compartment: (**Left Panel)** Comparison between HIV-seropositive patients with and without active HIV viremia based on HIV viral load below or above 50 copies/ml; (**Middle Panel**) Correlation with HIV viral load in log copies/ml among HIV-viremic patients (i.e. >50 copies/ml) (middle); (**Right Panel**) Comparison between HCV-monoinfected patients, HCV/HIV-coinfected patients with undetectable HIV viremia and HIV-monoinfected subjects with undetectable HIV viremia.
